# Healthcare technologies, quality improvement programs and hospital organizational culture in Canadian hospitals

**DOI:** 10.1186/1472-6963-13-413

**Published:** 2013-10-13

**Authors:** Rajesh K Tyagi, Lori Cook, John Olson, James Belohlav

**Affiliations:** 1 Department of Logistics and Operations Management, HEC Montréal, 3000, Chemin de la Côte-Sainte-Catherine, Montréal, Québec H3T 2A7, Canada; 2 Department of Management, DePaul University, 1 East Jackson Blvd, Chicago, IL 60604, USA; 3 Operations and Supply Chain Management, University of St. Thomas, 2115 Summit Ave, St Paul, MN 55115, USA

## Abstract

**Background:**

Healthcare technology and quality improvement programs have been identified as a means to influence healthcare costs and healthcare quality in Canada. This study seeks to identify whether the ability to implement healthcare technology by a hospital was related to usage of quality improvement programs within the hospital and whether the culture within a hospital plays a role in the adoption of quality improvement programs.

**Methods:**

A cross-sectional study of Canadian hospitals was conducted in 2010. The sample consisted of hospital administrators that were selected by provincial review boards. The questionnaire consisted of 3 sections: 20 healthcare technology items, 16 quality improvement program items and 63 culture items.

**Results:**

Rasch model analysis revealed that a hierarchy existed among the healthcare technologies based upon the difficulty of implementation. The results also showed a significant relationship existed between the ability to implement healthcare technologies and the number of quality improvement programs adopted. In addition, culture within a hospital served a mediating role in quality improvement programs adoption.

**Conclusions:**

Healthcare technologies each have different levels of difficulty. As a consequence, hospitals need to understand their current level of capability before selecting a particular technology in order to assess the level of resources needed. Further the usage of quality improvement programs is related to the ability to implement technology and the culture within a hospital.

## Background

Canadian healthcare has been the subject of significant discussion from a variety of perspectives. One area of discussion has focused on the level of spending. Canada’s healthcare spending in 2010 was estimated to be $183 billion, which resulted in a per capita expenditure of $4,478 [1]. Even being one of the top spenders among the OECD countries, Canada was ranked only 25^th^ in a comparative study with 33 European nations [2]. Regardless of where Canada’s healthcare spending falls relative to other countries, considerable consequences have been identified for the Canadian healthcare system. For example, Ontario, one of the Canadian provinces, is facing a situation in 2015 where about 70% of tax revenues will be consumed by healthcare costs [3]. The impact of these recent observations was foreshadowed in an earlier study, which reported that a vast majority of Canadian hospital executives felt that their financial situation was insufficient to maintain their current levels of service [4].

Even though cost is an important issue in the healthcare discussion in Canada, the results of those expenditures, the level of quality, has been a topic of many debates and comparisons [5]. One important area in the quality of care discussion that has received attention is the occurrence of adverse events [6]. Several views for resolving the cost-quality dilemma confronting the Canadian healthcare system have been provided. One perspective for increasing service quality within existing cost constraints has focused on enhancing innovation, developing healthcare information technologies and fostering an appropriate culture [3]. Another approach focused on the use and implementation of quality improvement programs throughout Canada [7]. A common denominator in the preceding viewpoints is the creation of managerial or organizational capabilities within hospitals. Indeed it has been pointed out that the underlying “performance improvement capability” in hospitals is a key factor leading to the recognition and adoption of improvements that lead to higher levels of performance [8].

All of the preceding perspectives for improving Canadian healthcare have been shown to be effective measures in previous research. The capability to implement healthcare technologies has been shown to improve performance within hospitals. Healthcare technologies have been shown to reduce medical errors [9] and to improve safety [10,11]. In addition to healthcare technologies, hospitals are enhancing internal processes by employing quality improvement programs. Some quality improvement programs have been created within the health care setting while other quality initiatives have come from outside of healthcare from areas such as manufacturing or service industries [12]. In general, increasing quality has been shown to improve operational performance [13]. Specifically, the usage of improvement programs has been related to various types of performance within hospitals. Positive relationships have been found relative to efficiency improvement and optimizing quality of care [13], turnover intention [14], waiting and lead times [15] and safety [16]. Finally, organizational culture and values have been shown to impact quality of care and reduce medical errors [17], hospital errors [18] and safety [19].

While individual studies have shown that each of the preceding variables has been related to improvements in hospital performance, a literature search revealed little about understanding the interrelationships among the variables. That is, even though healthcare technologies and quality improvement programs arguably have shown positive relationships to hospital performance, it is not clear whether there is a relationship between the ability to implement healthcare technologies and quality improvement programs. In addition, organizational culture within hospitals has shown a positive relationship to hospital performance; however, there is no clear understanding of how it relates to healthcare technologies and quality improvement programs within hospitals. Some research findings have indicated that culture functions as a mediating variable. The mediating nature of culture has been identified in both healthcare organizations [20] as well as other types of organizations [21,22].

The purpose of this study is to examine how the capability to implement healthcare technologies relates to improvement program usage within Canadian hospitals. Further, we will examine the role of organizational values and culture as a mediating variable within hospitals.

## Methods

### Procedures and participants

The data for this study was obtained from a self-report questionnaire sent to individuals in hospitals throughout Canada. Using the database provided by the Canadian Health Association for year 2009-2010, a total of 592 hospitals were targeted within the 12 provinces. The target group in the study focused on decision makers within hospitals in the mid to upper management levels such as: the Chief Executive Officer, Chief Medical Officer, Chief Operating Officer, or director of clinical quality, which was later modified during the course of ethics review process. A second mailing was sent out to gather data from hospitals that did not respond to the initial mailing. Overall, responses were received from 134 hospitals, resulting in an effective hospital response rate of 22.6 percent. In comparing the responses from the first mailing to the second mailing, no statistically significant differences were observed in the data. The questionnaire was distributed through HEC in Montreal, Canada in both English and French versions using the online survey management platform Unipark, which assured anonymity of the respondents.

### Ethics

The current project was initially approved by the ethics committee at HEC Montréal, which required complete anonymity for the respondents. The questionnaire also had to go through a final ethics approval process at the local level, which varied by each of the Canadian provinces. In a centralized structure, for example in Alberta, the questionnaire was sent to the central authority for the ethics approval. In a decentralized or region-based structure, for example in Quebec, the questionnaire was sent directly to the initially selected individual in the hospital; however, many of these individual hospitals also required an additional ethics approval. It should be noted that in the course of the final ethics approval that some of the individuals originally selected to be part of the sample were modified as a result of the ethics approval process to an individual who was deemed to be more appropriate relative to the information that was sought in the questionnaire.

### Instrument

The questionnaire used in the study consisted of three sections. Two of the sections gathered data on the usage of healthcare technologies (independent variable) and quality improvement programs (dependent variable), which were measured using a dichotomous choice format. The third section consisted of organizational culture questions using a five point Likert item scale (mediating variable). The questionnaire consisted of English and French versions so that an appropriate questionnaire could be matched to a particular population (English speaking versus French speaking individuals), which was included as a control variable. The full questionnaire scale and a short description of its development process are presented in the Appendix A.

### Rasch model analysis

The initial part of this study utilized Rasch model analysis (RMA) [23]. RMA is described more generally as a latent trait analysis. Using this kind of analysis, one tries to define an underlying factor that cannot be observed or measured directly from other observable variables. In this study, RMA will attempt to define technology implementation, the independent variable in this analysis [24]. The model construction in this analysis is detailed in Appendix B.

RMA was utilized in this analysis because of several noteworthy features that it possesses. First, RMA identifies whether a hospital’s capacity to adopt healthcare technology is unidimensional in nature. If it is, then the independent variable would be interpreted as a underlying technological capability of a hospital. Second, RMA converts all of the original data into true interval scale data [25]. Thus, the dichotomous data collected in the questionnaire are changed into equal units by means of logarithmic transformation. A third characteristic of RMA is the requirement of invariance. When invariance exists, it simply means that the difficulty of healthcare technology items can be assessed independently of a hospital’s capability of adopting or implementing healthcare technologies. If the data fit the Rasch model requirements, the results are referred to as being sample independent. Thus, the results of this study would be applicable to other hospitals that are considered to be part of the same population even though they were not part of the present analysis [26,27]. A final feature of RMA is that it enables the same dataset to both estimate and test solutions [23,26,27]. Additional technical discussions on Rasch model analysis can be found in the general RMA literature [25,28].

### Mediation analysis

In general, mediation analysis views whether a process exists that underlies the relationship between an independent variable and a dependent variable. That is, a mediating relationship occurs when another variable plays some elemental role that influences the relationship between the other two variables. In this study, the role of organizational culture is examined as a mediating factor, which influences the relationship between the ability to implement technology and the use of improvement programs within a hospital.

## Results

### Measurement properties of healthcare technologies

The first aspect of the present analysis examines whether the Rasch model can be used with the data from Canadian hospitals in this study. More specifically, the initial part of the analysis concentrates on whether the data is consistent with the expectations of the Rasch model. When the data corresponds with the Rasch model, it signifies that a fundamental underlying dimension exists that is shared by the various healthcare technologies examined. Winsteps version 3.63.2 [29] was used to analyze the data.

Linacre [30] proposes a sequential method to determine the agreement of the data with the prespecified Rasch requirements. The first step identifies whether contradictions are present in the latent variable by means of point measure correlation, r_pm_. The first aspect of the analysis observes if an individual healthcare technology relates to the latent variable as a whole. This part of the analysis is concerned principally with the sign of the correlation instead of the magnitude of the relationship. Overall, a positive sign would indicate consistency between an individual healthcare technology and the latent variable. A negative sign or a value close to zero would indicate an item that is not consistent with the model. As is shown in Table 1, all of the healthcare technologies except RFID and Barcode patients met the first step criteria. Thus, all of the healthcare technologies with the exception of RFID and Barcode patients are retained in the model.

**Table 1:** Healthcare technology variables by level of difficulty

**Healthcare technology variables**	**Difficulty measure**	**SE**	**Infit MNSQ**	**Infit ZSTD**	**Outfit MNSQ**	**Outfit ZSTD**	**r**_***pm***_
							
Barcode medications	2.19	0.60	0.99	0.2	0.45	-0.2	0.23
Barcode medical charts	1.87	0.53	1.03	0.2	1.17	0 .5	0.17
Electronic medical records (EMR)	0.92	0.36	0 .92	-0.3	0.57	-0.7	0.38
Computerized reminder systems	0.78	0.36	1.12	0.6	0 .95	0 .1	0.26
Electronic nursing notes	0.53	0.34	0.95	-0.2	1.50	1.1	0.34
Computerized order sets	0.42	0.33	0.96	-0.1	0.80	-0.3	0.38
Electronic pharmacy orders	0.42	0.33	0 .98	0.0	0.75	-0.4	0.40
Medical automated recording system (MARS)	0.42	0.33	1.06	0 .4	1.32	0 .8	0.33
Computerized treatment protocols	0.22	0.31	0.95	-0.2	0.90	-0.1	0.41
Computerized physician order entry (CPOE)	0.13	0.30	0.97	-0.1	0 .72	-0.7	0.43
Automated medical administration	-0.05	0.29	0.95	-0.2	0.65	-1.0	0.47
Computerized clinical guidelines	-0.14	0.29	1.13	0 .9	1.50	1.5	0.32
Computerized education reference tool	-0.30	0.28	1.04	0 .3	1.23	0 .8	0.39
Automated medical dispensing devices	-0.60	0.27	1.00	0.1	1.18	0 .7	0.45
Picture archive and communication system (PACS)	-0.94	0.26	0.90	-0.9	0.75	-1.2	0.57
Diagnostic Imaging	-2.06	0.20	1.00	0 .1	1.02	0.2	0.53
Benchmarking	-3.80	0.30	1.02	0 .2	1.06	0 .3	0.47
**Excluded healthcare technology variables**	**Measure**	**SE**	**Infit MNSQ**	**ZSTD**	**Outfit MNSQ**	**ZSTD**	**r**_***pm***_
RFID	3.15	1.01	1.03	0.40	1.02	0.70	-0.02
Barcode patients	2.55	0.73	1.05	0.30	0.92	0.40	-0.01
Barcode lab reports	-0.13	0.93	1.22	1.30	1.64	1.50	0.20

The second aspect of the analysis views how much useful information each individual healthcare technology contributes to the overall model. In RMA, the variance that is measured is the difference between the expected score and the actual data value. When a Rasch model is able to be constructed, then all systematic variance is explained in one dimension [30]. RMA uses two statistics, outfit and infit statistics, to assess the variance and ultimately its contribution to the overall model. The outfit statistic views variations from expected values for healthcare technologies that are distant from a particular hospital’s capability location on the Rasch continuum. The outfit statistic is a conventional sum of squared standardized residual. The infit statistic views variations from expected values for healthcare technologies that are relatively near a particular hospital’s capability location on the Rasch continuum. The infit statistic is an information-weighted mean square fit statistic, which reduces the inordinate impact of outlier responses [31]. Both the outfit and infit statistics, reported in Table 1, are generally considered to meet the Rasch model requirements when a particular healthcare technology’s mean-square exhibits a value within a range of 0.5-1.5 [32,33] and is normally distributed (i.e. z-score = ±2 s.d.). With the exception of Barcode lab reports, all of the remaining healthcare technology variables fell within the mean square and standard deviation guidelines. As a result, the preceding healthcare technology variable, Barcode lab reports, was removed from the analysis in order to create the final model. When the data corresponds to the expectations of the Rasch model, the resulting outcomes are considered to be both sample and scale independent.

The final aspect of this analysis investigates whether there is another competing explanation for the data a principal components analysis of the Rasch standardized residuals. Linacre [29] suggests that one method for identifying an alternate explanation for the data other than can be done by considering the magnitude of explained variance for the Rasch model relative to the unexplained variance in the 1st contrast, which would indicate the potential existence of an alternative explanation. Linacre notes that a result with an explained variance that is greater than 60% with an accompanying unexplained variance of less than 5% in the 1st contrast as a model that provides a suitable Rasch explanation of the data. In the present study, the variance explained by the Rasch measures was 63.4% while the unexplained variance in the 1st contrast was 2.1%. Given this result, The Rasch explanation of the data appears to be the most relevant explanation.

Along with the validity of the model, an assessment was made of the model’s reliability. That is, we viewed whether the healthcare technologies included in the model were able to produce an internally consistent measure. From the previous discussion, we were able to independently measure the reliabilities of healthcare technologies and hospitals. The Rasch reliability for hospitals is 0.60, which results in a Cronbach α of 0.70, and a Rasch reliability for healthcare technologies of 0.93. The reliability of the present Rasch model indicates that it is an internally consistent measure. The overall results would allow one to conclude that a unidimensional latent trait is present, which will be referred to as the technological capability within a hospital.

As part of the Rasch analysis, we can compare the response patterns for the two versions of the questionnaire, English and French. This aspect is called a Differential Item Functioning analysis or a DIF analysis. In this study, there were no differences observed in responses on English and French questionnaires for the healthcare technologies comprising the independent variable.

### Assessing outcomes

Since the data were found to be consistent with the Rasch model requirements, two types of outcomes can be analyzed. The first outcome to be viewed is the difficulty of healthcare technologies themselves. The second outcome to be examined is the ability of hospitals to implement healthcare technologies in relation to the number of quality improvement programs that are being used. In addition, we will view whether the culture in the hospital mediates the relationship of the ability to implement healthcare technologies and the usage of quality improvement programs.

Since the independent variable was transformed into interval data in the process of creating the Rasch model, it would be useful to further examine the healthcare technologies. Table 1 displays the difficulty of each healthcare technology. The difficulty measures represent how hard or how easy a particular healthcare technology is to implement. Negative scores represent technologies that are easier to accomplish and positive scores represent technologies that are more difficult to accomplish. Thus, Table 1 presents a hierarchy of difficulty among the different healthcare technologies, where the technologies at the top of Table 1 are more difficult to accomplish than those at the bottom. Specifically, we will assess the statistical differences among the various healthcare technologies using a z-score. To do so, we will use the difficulty measures and their respective standard error scores from Table 1. The z-score was calculated using the following formula: (Difficulty Measure_a_- Difficulty Measure_b_)/(1.5*(Standard Error_a_-Standard Error_b_)). If we view the 80^th^ percentile healthcare technology (Electronic medical records) and the 20^th^ percentile healthcare technology (Picture archive and communication), we observe that a statistically significant difference in difficulty (z =2.00) exists. This result identifies that healthcare technologies at the 80^th^ percentile or higher are statistically more difficult to accomplish than are healthcare technologies at the 20th or lower percentile in Canadian hospitals.

### Regression analysis

The next aspect of the analysis will examine the ability of hospitals to implement healthcare technologies with respect to the number of quality improvement programs that they employ. In this part of the analysis, we utilize regression analysis using the Rasch ability score for each hospital (not reported) and language as a control variable. The regression analysis first viewed the influence of the control variable to assess whether any differences were present within hospitals. Language of the respondent in the organization was not found to have any statistically significant relationship with quality improvement program usage. The control variable was removed from further analysis. Next, the technological ability scores for the hospitals developed by the Rasch analysis were entered into the regression analysis. The results from the final model indicate the Rasch technological ability score is significantly correlated with the number of improvement programs adopted, F (1, 94) = 30.47, p < .01. The r^2^ = 0.245 indicates that over 24 percent of the total variance in the number of improvement programs adopted is explained by the Rasch model ability score.

### Mediation analysis

**Figure 1:**
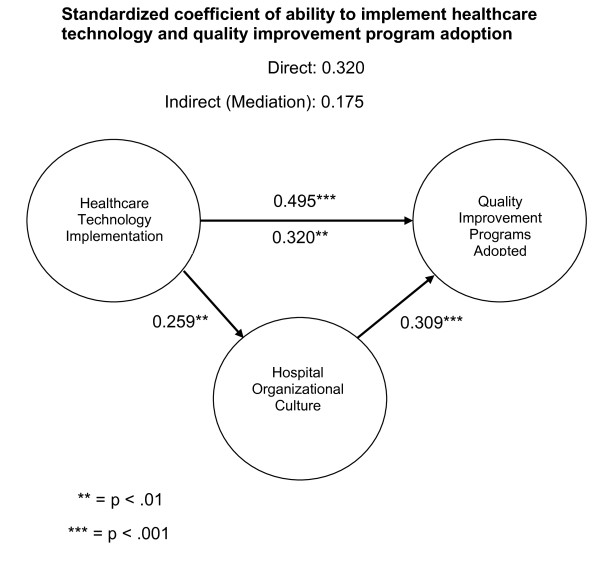
Mediation model.

The concluding aspect of the analysis will examine the mediating role of hospital culture. In order to assess whether mediation exists a procedure presented by Kenny et al. [34] was employed. MedGraph-I version 2.0 [35] was used to assess mediation. A preliminary requirement for mediation is a statistically significant correlation among the variables. From Figure 1, one identifies that statistically significant relationships exist among all of the variables. To assess if significant mediation is present, we will apply Sobel’s z statistic. In this study, this statistic is statistically significant (z = 12.469, p < .001), indicating that a meaningful mediating relationship exists.

After the mediating variable was entered into the analysis, hospital culture, the association between the ability to implement healthcare technologies and quality improvement program usage remained significant (r = 0.320 p < .01). This result indicates that partial mediation is present. Partial mediation means that hospital culture imparts a partial intervention or indirect effect. Thus, the overall correlation in this study consists of a direct effect from the ability to implement healthcare technologies on hospital performance related to quality improvement program usage (r = 0.320) and an indirect effect that goes through the mediating variable (r = 0.175), organizational culture. The indirect effect in this study accounts for 35% of the overall relationship in this study. That is, the ability to implement healthcare technologies accounts for 65% of quality improvement program adoption or usage and the hospital culture accounts for 35% of quality improvement program adoption or usage.

## Discussion

Several noteworthy findings arose from this study that is relevant to Canadian hospitals. First because the Rasch model was able to be successfully applied to the data in this study, it signified that an underlying or latent relationship exists, which is referred to as technological capability. What creates technological capability? Existing literature suggests that complementarity may play a role where one particular technology may develop some increasing influence when linked with other technologies [36]. Thus, a hospital that implements greater numbers of technologies may produce a distinct competence within its operations [37,38]. Thus, Canadian hospitals that have greater levels of technological capability were able to more often successfully implement healthcare technologies within their hospitals. Other hospitals with less capability were able to implement fewer technologies within their organizations.

The second finding related to the healthcare technologies themselves. Specifically, individual healthcare technologies vary in their level of difficulty for Canadian hospitals, which essentially formed a hierarchy of difficulty. There was a statistically significant difference between technologies at the top of the hierarchy, more difficult, shown in Table 1 relative to those at the bottom of the hierarchy, less difficult. That is, Barcode medications and Barcode medical charts as well as Electronic medical records were more difficult to implement than benchmarking, diagnostic imaging and picture archive and communication. What does this mean from a practical perspective? Assume a hospital has been able to accomplish these 3 lowest healthcare technologies. Let us further assume that the hospital next wants to implement a computerized physician order entry system (CPOE) into its operations. Since the difficulty measure in Table 1 now represents a true interval scale, it can be interpreted just like we would use an ordinary ruler. Since CPOE is 1 unit away from what the hospital has already accomplished, undertaking the implementation of a CPOE system would be twice as difficult as what it has already accomplished. If they chose to implement an electronic medical records system (EMR) it would be almost three times as difficult to accomplish because it is almost 2 units away from picture archive and communication, which it has already accomplished.

The third finding in this study is that a statistically significant relationship exists between a hospital’s technological capability and the number of quality improvement programs that it utilizes. The current study identifies that technological capability is a cumulative relationship because the Rasch model is cumulative in nature. This finding is consistent with the general management literature, which suggests that implementation of technologies may both be influenced by cumulative learning, expert knowledge, and unique insights that may lead to their successful adoption within organizations [39-41]. There may be certain knowledge based competencies relating to implementation that are created as a hospital increases its capability. The cumulative learning may be a function of expert knowledge developed by a hospital for overcoming obstacles, specific hospital processes or organizational structures. This particular finding implies that there may be a common factor influencing the implementation of technology as well as quality improvement programs.

The final finding relates to the culture within the hospital. The term culture has been used in several ways in the healthcare literature. In this particular study, culture relates to a more all-encompassing description of factors affecting how a hospital operates or its overall performance. The previous discussion highlighted technological capability as being associated with increasing quality improvement program usage. However the culture within a hospital was also associated with increasing quality improvement program usage. This result implies that something exists in the culture separate from cumulative learning of technological capability. For example, a greater emphasis on integrating safety in hospital processes and structure or the use of cross-functional teams throughout a hospital may influence quality improvement program adoptions.

### Research limitations

This study has several significant limitations. First this study was based upon the responses of hospitals located in Canada, which exist largely as not-for-profit organizations. As a result, the current findings may not be applicable to all types of hospitals. Second, while we believe that our sample was representative of Canadian hospitals, it may have contained respondent bias. Bias was examined from several different perspectives. No significant differences were observed: 1) in response patterns between the first and second mailings and 2) between English and French versions of the questionnaire. Third, Like van Lent et al. [42] and Yasin et al. [43], we ultimately sent the questionnaire to only one individual in a hospital. Using this method can lead to single-source measurement bias. Based on the ethics reviews boards, we believe that respondents selected had the appropriate expertise on healthcare technologies, quality improvement programs and knowledge of the operational activities in their particular hospital setting. Fourth, there may be limitations in the data itself. The healthcare technologies and quality improvement programs included in this analysis may have excluded some noteworthy technologies or programs. In addition, the questionnaire did not observe how widely healthcare technologies or quality improvement programs were dispersed throughout a hospital only whether a hospital employed a particular technology or program. Finally, the research design in this study used a cross-sectional analysis, which is principally descriptive in nature.

## Conclusions

Although there have been a variety of articles about the efficacy of different healthcare technologies, there have been few studies that have viewed a broad range of healthcare technologies [44]. Rather most research focuses on only one or two technologies. As a consequence, many healthcare professionals are left wondering about the best choices within a resource constrained environment. The precision of any results or conclusions are limited by how good the measuring stick is. In this study, we are able to develop a measuring stick that concurrently allows the comparison of multiple healthcare technologies. While the findings of this study are useful, they present only a starting point for hospital decision making. An equally important activity involves identifying the capability level of a particular hospital because it is a crucial factor in determining the necessary level of resources in order to select and implement a specific healthcare technology. Finally, further research should be undertaken to better understand the common processes that underlie the capability for implementing healthcare technologies and the adoption of quality improvement programs.

## Appendix A

The questionnaire used in the study gathered three types of hospital organization data. One part gathered data on the usage of 20 healthcare technologies, which served as the independent variable. A second part gathered data on the usage of 16 quality improvement programs, which served as the dependent variable. The third part consisted of 63 hospital organization culture questions, which served as the mediating variable.

The 20 healthcare technologies investigated in this study were: Automated Medical Administration, Automated Medicine Dispensing Devices, Barcode-Charts, Barcode-Lab Reports, Barcode-Medication, Barcode-Patients, Benchmarking, Computerized Clinical Guidelines, Computerized Education Reference Tool, Computerized Physician Order Entry (CPOE), Computerized Order Sets, Computerized Reminder Systems, Computerized Treatment Protocols, Diagnostic Imaging, Electronic Medical Records (EMR), Electronic Nursing Notes, Electronic Pharmacy Orders, Medical Automated Recording System (MARS), Picture Archive and Communication System (PACS), RFID. The number of healthcare technologies implemented in the responding organizations ranged from 0 to 12 different technologies with the median number being 3 healthcare technologies.

The 16 quality improvement programs considered in this study were: Balanced Scorecard, Cross-functional Teams, Customer Relationship Management, Employee Recognition Programs, Employee Suggestion system, Internal Quality Award Program, ISO/TS certified, Lean Organization, Pay Bonus Plans, Safer Healthcare Campaign, Six Sigma, Statistical Process Control (SPC), Supplier or other external awards, Supply Chain Management, Team Quality Award, Voice of the Customer. The number of quality improvement programs implemented in the responding organizations ranged from 0 to 12 different programs with the median number of quality improvement program adoptions being 3.

The final variable organization culture within a hospital was created by summating items from an organization culture scale that was validated in an earlier study [19]. The response format of the organization culture scale is a 5-point Likert-type of scale (1 = never true to 5 = always true). The questions defining culture in hospitals that were validated in an earlier study had a Cronbach α of 0.94 in the current study of Canadian hospitals.

Items in the organizational culture questionnaire were generated, tested and validated according to a rigorous process. The initial item pool was created with several groups of individuals who were familiar with the MBNQA criteria. Specifically, items were generated according to the core values as proposed in MBNQA criteria [45,46]. The initial version of the questionnaire was pretested on MBA students. A refined questionnaire was further validated on a group of award winning organizations from the Illinois state quality award program, the Lincoln Award. Award winning organizations were made up of several economic sectors including healthcare organizations, manufacturing organizations, service organizations, small business organizations, education organizations and not-for-profit organizations. From an initial 63 item scale 57 items were selected based on RMA as representing organizational culture in hospitals. The complete questionnaire development process is described in an earlier study [19].

The items on organizational culture within the questionnaire included the following set of questions:

1. Our leaders set clear expectations for their employees.

2. Our leaders encourage employees to contribute to the organization.

3. Our leaders develop strategies with a customer or patient focus.

4. Our leaders inspire employees.

5. Our leaders make decisions based upon actual results.

6. Our leaders encourage employees to be innovative.

7. My organization provides employees with opportunities for personal learning through education, training, and other means for continuing growth.

8. My organization strives to improve our products or services.

9. My organization emphasizes the sharing of knowledge throughout the organization.

10. My organization provides training based upon organizational needs and priorities.

11. My organization resolves complaints by “making things right” for our customers or patients.

12. My organization uses flexible work practices based upon both workplace and home life needs.

13. My organization goes beyond simply meeting local, state and federal laws and regulatory requirements.

14. My organization is concerned with employee satisfaction and well-being.

15. My organization provides employees with recognition beyond just traditional compensation.

16. My organization bases pay upon an individual’s knowledge and skills.

17. My organization provides opportunities for the personal development of its staff.

18. My organization utilizes measures that provide useful results.

19. My organization focuses on reducing the time it takes to get a product or service to a customer or patient.

20. My organization empowers its employees.

21. My organization identifies new ways to improve our performance.

22. My organization integrates its strategic objectives throughout the organization.

23. My organization has a strong future orientation.

24. My organization participates in benchmarking programs that compare our practices and performances with other organizations.

25. My organization focuses on managed levels of growth.

26. My organization collects information so that decisions can be made

27. Performance in my organization focuses on innovation that leads to improvements in our products, services, and operations.

28. Performance in my organization focuses on reducing time in order to enhance quality or cost.

29. Performance in my organization focuses on using measures that “lead” actual performance so that changes can be made to our operations before adverse impacts become visible.

30. Performance in my organization focuses on utilizing competitive comparisons to improve our operations.

31. Performance in my organization focuses on market share growth.

32. Performance in my organization focuses on anticipating changes in the market.

33. Performance in my organization focuses on allocating resources based upon changes in competition or technology.

34. Performance in my organization focuses on differentiating our products and services from our competition.

35. Performance in my organization focuses on making changes in our operations based upon our learning.

36. Performance in my organization focuses on trying to balance the needs of our stakeholders (i.e. customers, patients, employees, suppliers, the public and the community).

37. Performance in my organization focuses on developing external partnerships with customers, patients or suppliers.

38. Performance in my organization focuses on improving existing measures to better meet organizational goals.

39. Performance in my organization focuses on involving non-managerial and non-supervisory workers in regularly scheduled meetings to discuss work-related problems such as working conditions, health and safety, technology and improving specific tasks.

40. Operating concerns in my organization emphasize developing an awareness of technology and competitor offerings.

41. Operating concerns in my organization emphasize the capacity for rapid change and flexibility.

42. Operating concerns in my organization emphasize internal partnering.

43. Operating concerns in my organization emphasize the conservation of environmental resources and waste reduction.

44. Operating concerns in my organization emphasize anticipating the adverse environmental or social impacts of our operations.

45. Operating concerns in my organization emphasize incorporating “best practices” into our operations.

46. Operating concerns in my organization emphasize activities that focus on improving the organization as a whole.

47. Operating concerns in my organization emphasize actively makes information available to the public on organizational ethics, public health, safety and the environment.

48. Operating concerns in my organization emphasize creating partnerships with other organizations on issues relating to public responsibility and citizenship.

49. Operating concerns in my organization emphasize measuring key organizational processes.

50. Operating concerns in my organization emphasize aligning our resources for faster response to our customers or patients.

51. Operating concerns in my organization emphasize aligning strategies with our organizational needs.

52. Operating concerns in my organization emphasize innovation that builds upon existing knowledge.

53. Operating conc erns in my organization emphasize developing a long-term commitment to our stakeholders (i.e. customers, patients, employees, suppliers, the public and the community).

54. Operating concerns in my organization emphasize customer or patient satisfaction and retention.

55. Operating concerns in my organization emphasize eliminating adverse impacts on our stakeholders (i.e. customers, patients, employees, suppliers, the public and the community).

56. Operating concerns in my organization emphasize removing obstacles to improvements.

57. Operating concerns in my organization emphasize ethical behavior in all stakeholder relations.

## Appendix B

The data were initially analyzed using Rasch Model Analysis (RMA), which was developed by Georg Rasch [23] for analyzing dichotomous data. It should be noted that the Rasch model is a prespecified model, which exists as a unidimensional, linear measure. Specifically, the analysis in this study views whether the data are consistent with the Rasch requirements.

RMA attempts to identify underlying factors that cannot be measured directly from other observable variables. Thus, the independent variable in this analysis is latent rather than an observed variable [24]. It does so by utilizing two basic parameters, which are referred to as the difficulty, δ, and the ability, β, components. The most familiar version of the model is depicted by the following equation:

$$p=\exp\;\left(\beta –\delta \right)/\left[1+\exp\;\left(\beta –\delta \right)\right]$$

For the present study, it is proposed that a healthcare technology’s difficulty, δ, and a hospital’s ability to implement a healthcare technology, β, can be located along the same latent dimension or variable. One would expect that healthcare technologies would present differing degrees of effort for hospitals trying to implement these technologies, where more complex or difficult technologies are accomplished less frequently. Likewise, one would expect that hospitals have differing capacities in being able to adopt or implement various healthcare technologies, where more capable hospitals are able to implement greater number of numbers of technologies within their hospitals. In this study, RMA uses the difficulty parameter, δ, to locate healthcare technologies along a continuum of technological capability while conjointly locating hospitals based on their ability to implement healthcare technologies, β, along the same continuum.

## Competing interests

The authors declare that they have no competing interests.

## Authors’ contributions

RKT contributed in terms of the conception and design of the study, the coordination and acquisition of data, analysis of data, manuscript draft, and revision. LC contributed in terms of the conception and design of the study, manuscript draft, and revision. JO contributed in terms of the conception and design of the study, analysis of data, manuscript draft, and revision. JB contributed in terms of the conception and design of the study, analysis and interpretation of data, manuscript draft, and revision. All authors read and approved the final manuscript.

## Pre-publication history

The pre-publication history for this paper can be accessed here:

http://www.biomedcentral.com/1472-6963/13/413/prepub
